# Diagnostic value of video-oculography in progressive supranuclear palsy: a controlled study in 100 patients

**DOI:** 10.1007/s00415-021-10522-9

**Published:** 2021-03-21

**Authors:** Jessica Wunderlich, Anna Behler, Jens Dreyhaupt, Albert C. Ludolph, Elmar H. Pinkhardt, Jan Kassubek

**Affiliations:** 1 Department of Neurology, University of Ulm, Oberer Eselsberg 45, 89081 Ulm, Germany; 2 Institute of Epidemiology and Medical Biometry, University of Ulm, Ulm, Germany

**Keywords:** Video-oculography, Oculomotor function, Parkinsonism, Progressive supranuclear palsy, Diagnostic marker

## Abstract

**Background:**

The eponymous feature of progressive supranuclear palsy (PSP) is oculomotor impairment which is one of the relevant domains in the Movement Disorder Society diagnostic criteria.

**Objective:**

We aimed to investigate the value of specific video-oculographic parameters for the use as diagnostic markers in PSP.

**Methods:**

An analysis of video-oculography recordings of 100 PSP patients and 49 age-matched healthy control subjects was performed. Gain of smooth pursuit eye movement and latency, gain, peak eye velocity, asymmetry of downward and upward velocities of saccades as well as rate of saccadic intrusions were analyzed.

**Results:**

Vertical saccade velocity and saccadic intrusions allowed for the classification of about 70% and 56% of the patients, respectively. By combining both parameters, almost 80% of the PSP patients were covered, while vertical velocity asymmetry was observed in approximately 34%. All parameters had a specificity of above 95%. The sensitivities were lower with around 50–60% for the velocity and saccadic intrusions and only 27% for vertical asymmetry.

**Conclusions:**

In accordance with oculomotor features in the current PSP diagnostic criteria, video-oculographic assessment of vertical saccade velocity and saccadic intrusions resulted in very high specificity. Asymmetry of vertical saccade velocities, in the opposite, did not prove to be useful for diagnostic purposes.

**Supplementary Information:**

The online version contains supplementary material available at 10.1007/s00415-021-10522-9.

## Introduction

Progressive supranuclear palsy (PSP) is a four-repeat (4R) tauopathy predominated by subcortical pathology in neurons, astrocytes, and oligodendroglia associated with various clinical phenotypes [1]. For the clinical diagnosis, the movement disorder society (MDS)-supported PSP study group published new criteria in 2017 [2]. Ocular motor disorders, postural instability, hypo-/akinesis, and cognitive dysfunction are the core characteristics recorded in three levels of certainty each [2]. Further standardization of their application (e.g., by Multiple Allocations eXtinction [3]) was introduced by subsequent analyses. In the oculomotor field, vertical supranuclear gaze palsy contributes most to diagnostic certainty (level of certainty 1 in the oculomotor dysfunction domain: O1), followed by vertical saccades with reduced velocity (O2). Finally, an increased rate of square wave jerks (SWJ), the most common form of fixation interrupting saccadic intrusions (SI) with return saccades [4], or a defined inability of eyelid opening are features at certainty level 3 (O3) [2]. Although the specificity of criteria proposed by the National Institute of Neurological Disorders and Stroke and Society for PSP (NINDS-SPSP) was reported to be higher [5], the new, broader criteria are much more sensitive [6], as achieved not least by considering a wide range of various PSP entities [2, 7, 8] beyond the most common subtype of the “classical” PSP Richardson Syndrome (PSP-RS).

Biomarkers are needed to improve diagnosis and follow-up diagnostic procedures. Regarding oculomotor function, features of limited sensitivity but high specificity have been reported [9, 10]. In the original description in 1964, patients were reported to present mainly with downward gaze limitations [11], but this finding could not be reproduced in all studies [12]. An analysis of the clinical oculomotor parameters based on video-oculography (VOG) data is a promising way to evaluate their role in PSP diagnosis. Besides the analysis of parameters currently used for diagnosis, the examination of other oculomotor parameters like asymmetry of saccade velocities up- and downwards might also contribute to the diagnosis.

## Materials and methods

### Participants

In this retrospective study, we collected the data of 100 consecutive patients in our clinic who were diagnosed with PSP and 49 age-matched healthy controls who received an examination by video-oculography (VOG) of sufficient quality between the years 2005 and 2018. The PSP patients (55 PSP-RS, 42 PSP-Parkinsonism [PSP-P], three other subtypes) were re-classified according to the current criteria of the MDS working group. Detailed clinical and demographic data are summarized in Table 1, including sex, age, disease duration, PSP rating scale (PSPRS) scores, and Hoehn & Yahr scale. Patients enrolled before 2017 were reclassified according to the recent diagnostic criteria for PSP and its subtypes PSP-RS and PSP-P [2]. In addition to the oculomotor deficits classification, the patients were classified in the domains postural instability (P) and akinesia (A). Ocular signs must be associated with postural instability (P1, repeated unprovoked falls within 3 years or P2, tendency to fall on the pull test within 3 years) for a diagnosis of PSP-RS, while the ocular signs must be associated with A2 (parkinsonism levodopa resistant) or A3 (parkinsonism levodopa responsive) for a diagnosis of PSP-P. Here, 55 patients with probable PSP-RS were rated P1 (*n* = 37) or P2 (*n* = 18), and 42 patients with probable PSP-P were rated A2 (*n* = 23) or A3 (*n* = 19), respectively. Patients with additional diseases or health impairments, including diseases that could interfere with the oculomotor performance and other relevant neurological or psychiatric diseases, were not included.

**Table 1:** Demographic and clinical characteristics

	Controls (*n* = 49)	PSP (*n* = 100)	*p* (controls-PSP)	PSP-RS (*n* = 55)	PSP-P (*n* = 42)	*p* (PSP-RS-PSP-P)
Male:female ratio	34:15	53:47	0.057^a^	28:27	22:20	0.886^a^
Age, years	68; 66–70 (62–79)	71; 69–72 (48–83)	0.072^b^	69; 67–72 (48–83)	72; 70–75 (53–83)	**0.021**^**b**^
Disease duration, years	–	3; 3–4 (1–15)	–	2; 2–3 (0–10)	3; 3–4 (1–15)	0.102^b^
PSPRS, score	–	44; 39–48 (22–78)	–	42; 38–47 (22–76)	46; 42–54 (25–74)	0.215^b^
Hoehn & Yahr scale, score	–	4; 4–5 (2.5–5)	–	4; 4–5 (2.5–5)	4; 4–5 (2.5–5)	0.788^b^

Data of patients and controls are presented as median, 95% confidence interval of the median (minimum–maximum value). Significant results are shown in bold

*PSPRS *PSP-rating scale

^a^Differences in gender distribution between groups were investigated by Chi^2^ test

^b^Differences in other parameters between groups were investigated by Mann–Whitney-*U* test for independent samples

The study was approved by the ethics committee of the University of Ulm, Germany (Reference no. 76/20). In accordance with the documentation and notification obligations, all participants have agreed to the use of the data.

### VOG data acquisition and postprocessing

For the VOG measurements, two commercial eye-tracking systems were used. The EyeLink I^®^ system (SR Research Ltd., Osgoode, ON, Canada) was used until 2014 (*n* = 47 PSP patients), while later recordings were done with the EyeSeeCam^®^ system (EyeSeeTec GmbH, Munich, Germany) (*n* = 53 PSP patients). Both standardized and validated commercial systems allow fast and reliable tracking of both eyes with a very high accuracy; exactly the same paradigms have been executed in the standardized settings of our oculomotor laboratory. Two infrared cameras, which allow binocular eye tracking, are either mounted below (EyeLink I^®^) or above (EyeSeeCam^®^) the subject’s eyes. Prior to the eye movement measurements, the eye tracker system was calibrated for each subject to incorporate the individual geometric characteristics of each subject’s eyes into an accurate gaze point calculation. All examinations took place in our dedicated oculomotor laboratory, certified in accordance with DIN EN ISO 14971, as previously described in detail [13–17]. During the VOG examination, the participant sits in a chair in front of a white semi-cylindrical screen, so that the distance between eyes and screen is 150 cm. A chin rest is used to stabilize the head so that movement artifacts are minimized. Video recordings of the eyes are produced using miniature infrared cameras placed above the patient’s eyes.

Two paradigms were used: First, for smooth pursuit eye movement (SPEM), the instruction was to follow a point of light that is sinusoidally moving in horizontal or vertical direction with a frequency of 0.375 Hz and an amplitude of ± 20° with the eyes as accurately as possible. Second, in order to investigate visually guided reactive saccades, points lit up in pseudo-randomized sequence immediately following each other. The sequence consisted of 32 horizontal target movements with amplitudes of three times ± 5°, ± 10°, ± 15° and ± 40° each and four times ± 20° with a maximum span of ± 20° and 36 vertical target steps with amplitudes of four times ± 5°, ± 10°, ± 15° and ± 30° each and twice ± 20° with a maximum span of ± 15°. The participants were given the task of directing their gaze as quickly and again as accurately as possible to newly illuminated points and to keep it there until the next target point appears.

The VOG data were processed with the inhouse software system OculoMotor Analysis [18], based on MATLAB^®^ (The Mathworks Inc, Natick, Massachusetts, USA). The raw data were artifact-cleaned and calibrated by tracking a point of light moving slowly sinusoidally in horizontal and vertical directions by the participants. There were no systematic differences between the values of the left and right eye so that both could be averaged to a common value. In addition, the quality of the measurements was ensured by reviewing all images and excluding incorrect execution due to lack of cognitive or motor skills.

SPEM was examined on the combined vertical and horizontal achieved gain as a ratio of the actual velocity and the target velocity. To analyze the saccades, the averaged value of the vertical and horizontal latency was used as well as the gain, that was determined as a ratio of the primary saccade amplitude to the target amplitude, and the peak velocity, where the values for the upward and downward movements and, in the horizontal direction, the average value of the saccades to the left and right were evaluated. The SI rate was determined during the execution of horizontal saccades by adding all saccade amplitudes apart from the main saccade amplitude and saccade amplitudes below 2° and adjusting them to the time interval of one second.

### Statistical evaluation

IBM SPSS Statistics 25 (https://www.ibm.com/de-de/analytics/spss-statistics-software, Version 25.0) was used for statistical analysis. Group differences were tested for categorical variables with the Chi^2^ test or Fisher’s exact test, for continuous variables with the Mann–Whitney-*U* test for two groups and the Kruskall–Wallis-*H* test for more than two groups. In case of a significant Kruskall–Wallis-*H* test, post-hoc pairwise group comparisons were performed with the Mann–Whitney-*U* test and the *p* values were adjusted using the Bonferroni correction for multiple testing. Due to low sample size, patients with other clinical presentations than PSP-P or PSP-RS were excluded from subgroup analysis. Associations between different continuous parameters were investigated by scatter plots and the Spearman rank correlation coefficient. The associations between different groups and continuous parameters were investigated by Boxplots. Using the 5th and 95th percentile of the vertical peak saccade velocity, its asymmetry and the SI rate of the control group, limit values were defined. The determination of sensitivity and specificity to differentiate between patients and controls was performed from ROC analyses; this analysis was performed for the PSP sample as a whole and for the subgroups PSP-RS and PSP-P separately, respectively. The different oculomotor parameters were age corrected by linear regression based on the average age of the control group [15]. All statistical tests were performed at a two-sided level α = 0.05. Due to the exploratory nature of this study, all results from statistical tests have to be interpreted as hypothesis-generating.

## Results

### Oculomotor changes in PSP

As summarized in Table 2, all oculomotor parameters significantly differed between patients and controls. In detail, PSP patients achieved a lower gain of SPEM and saccades in all investigated directions; in addition, the latencies were prolonged, SI rate were increased, and the peak eye velocities were reduced in the horizontal plane and even more in both vertical directions (all, *p* < 0.001).

**Table 2:** Oculomotor characteristics

	Controls (*n* = 49)	PSP (*n* = 100)	*p*	Median of differences^b^
SPEM gain, %	0.80; 0.74–0.83 (0.33–0.96)	0.47; 0.42–0.51 (0.04–1.45)	** < 0.001**^a^	− 0.30; − 0.23/− 0.36
VGRS latency, ms	263.43; 246.67–277.57 (207.23–348.65)	372.46; 332.51–393.96 (213.83–1020.70)	** < 0.001**^a^	100.90; 73.13/134.98
VGRS (horizontal) gain, %	0.89; 0.87–0.90 (0.77–0.99)	0.73; 0.69–0.75 (0.13–1.12)	** < 0.001**^a^	− 0.17; − 0.13/− 0.21
VGRS (up) gain, %	0.79; 0.75–0.82 (0.58–1.04)	0.51; 0.48–0.60 (0.03–1.07)	** < 0.001**^a^	− 0.26; − 0.19/− 0.32
VGRS (down) gain, %	0.91; 0.89–0.96 (0.65–1.15)	0.61; 0.53–0.68 (0.05–1.05)	** < 0.001**^a^	− 0.32; − 0.24/− 0.42
VGRS (horizontal) peak eye velocity, °/s	433.47; 411.00–447.03 (264.48–517.35)	304.19; 262.46–326.49 (0.38–747.31)	** < 0.001**^a^	− 124.83; − 95.45/− 156.17
VGRS (up) peak eye velocity, °/s	367.55; 356.96–404.83 (209.27–618.32)	200.46; 154.08–234.53 (54.07–477.78)	** < 0.001**^a^	− 183.24; − 139.69/− 218.78
VGRS (down) peak eye velocity, °/s	401.71; 354.70–419.43 (224.68–524.88)	222.79; 170.15–266.22 (13.15–587.42)	** < 0.001**^a^	− 148.61; − 100.29/− 191.96
VGRS intrusion rate, °/s	3.67; 3.13–4.29 (1.14–10.60)	10.35; 9.02–12.09 (3.08–23.20)	** < 0.001**^a^	6.37; 5.13/7.73

Data are presented as median, 95% confidence interval of the median (minimum–maximum value). Significant results are shown in bold

*VGRS *visually guided reactive saccades, *SPEM *smooth pursuit eye movement

^a^Differences in the variables between groups were investigated by Mann–Whitney-*U*-test for independent samples

^b^Presentation of the median of differences and the 95% confidence interval

None of the investigated oculomotor parameters showed significant differences between the two most frequent subtypes PSP-RS and PSP-P (Supplemental Table 1).

### Vertical peak eye velocity

The 5th percentile of the vertical peak eye velocities of the age-corrected cohort of controls was defined as the cut-off value for determining whether a value achieved by the patient was regarded as pathological. The cut-off value for vertical movements was 236.09°/s; the limit was 241.52°/s for the upward movement and 243.25°/s for the downward movement. In 85 patients, measurements of the peak eye velocities of the upward and downward movement were available. While 51.8% of the PSP patients (*n* = 44) had pathological values for the upward and the downward movement, 29.4% (*n* = 25) were able to obtain higher velocities in both directions and thus fell into the group of patients with unremarkable measurements of vertical saccade velocity; finally, 18.8% (*n* = 16) showed pathological findings in only one of the two directions.

**Fig. 1:**
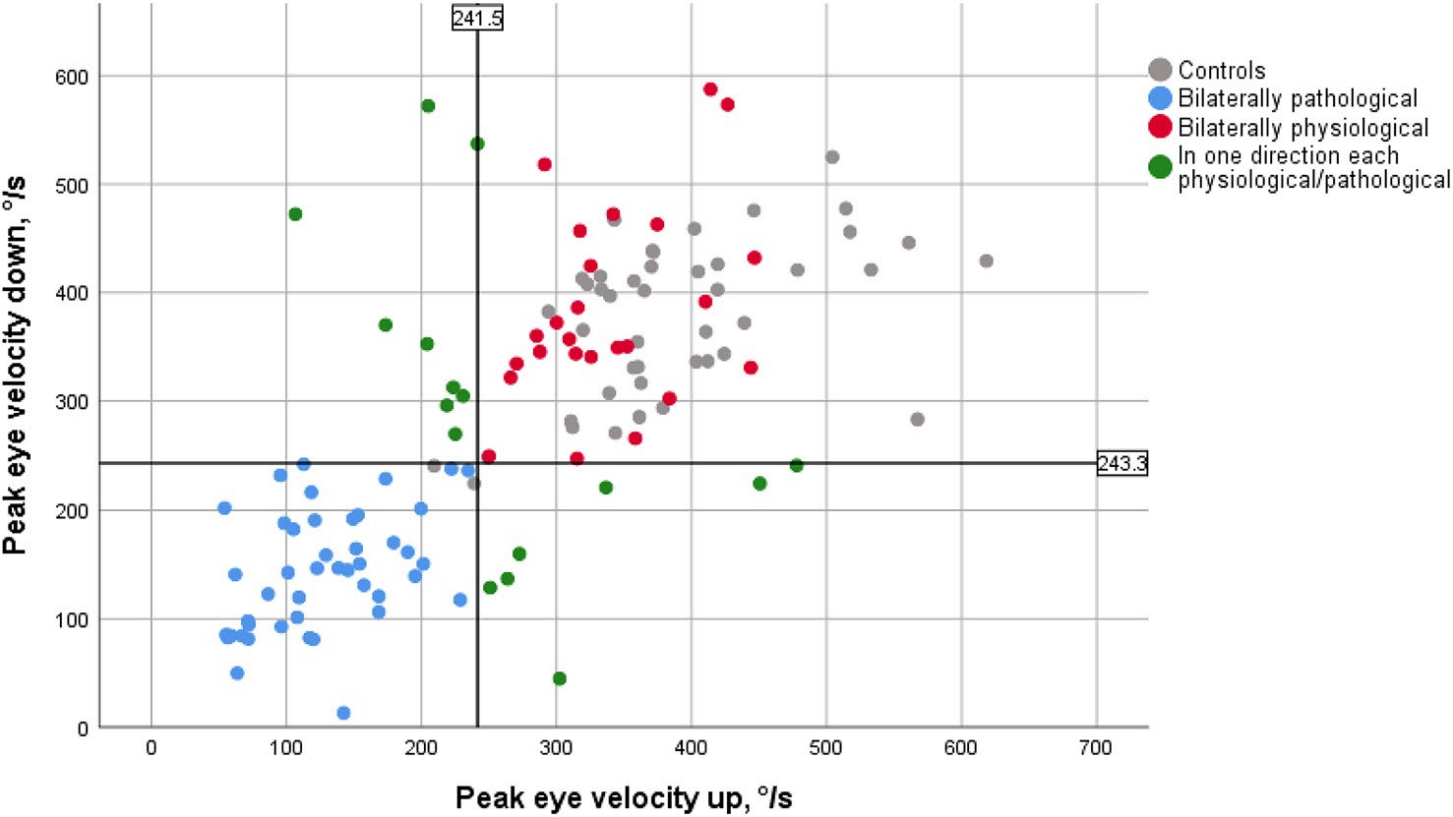
Peak eye velocities upward and downward. The values achieved by the control group (grey dots) are in the upper range of the patients’ results in both directions. The blue dots correspond to patients with pathological values in both directions. The patients with non-pathological values in both directions are shown as red dots, those in whom only one direction was considered to be pathological are shown as green dots. In the upward saccades, the patients reached only lower velocities than the controls, while the downwards velocities were higher in four patients. The black solid lines delineate the limits for upward and downward movement

There was a significant correlation between the saccade velocities of the two directions in the patients (*r* = 0.689, *p* < 0.001) and within the control group (*r* = 0.528, *p* < 0.001). The range of measurement values of the patients extended far beyond the range of the control group in both directions: the majority of the patients showed pathological values into the slower range, while single patients achieved higher velocities than the controls in the downward movement (Fig. 1).

### Rate of saccadic intrusions

**Fig. 2:**
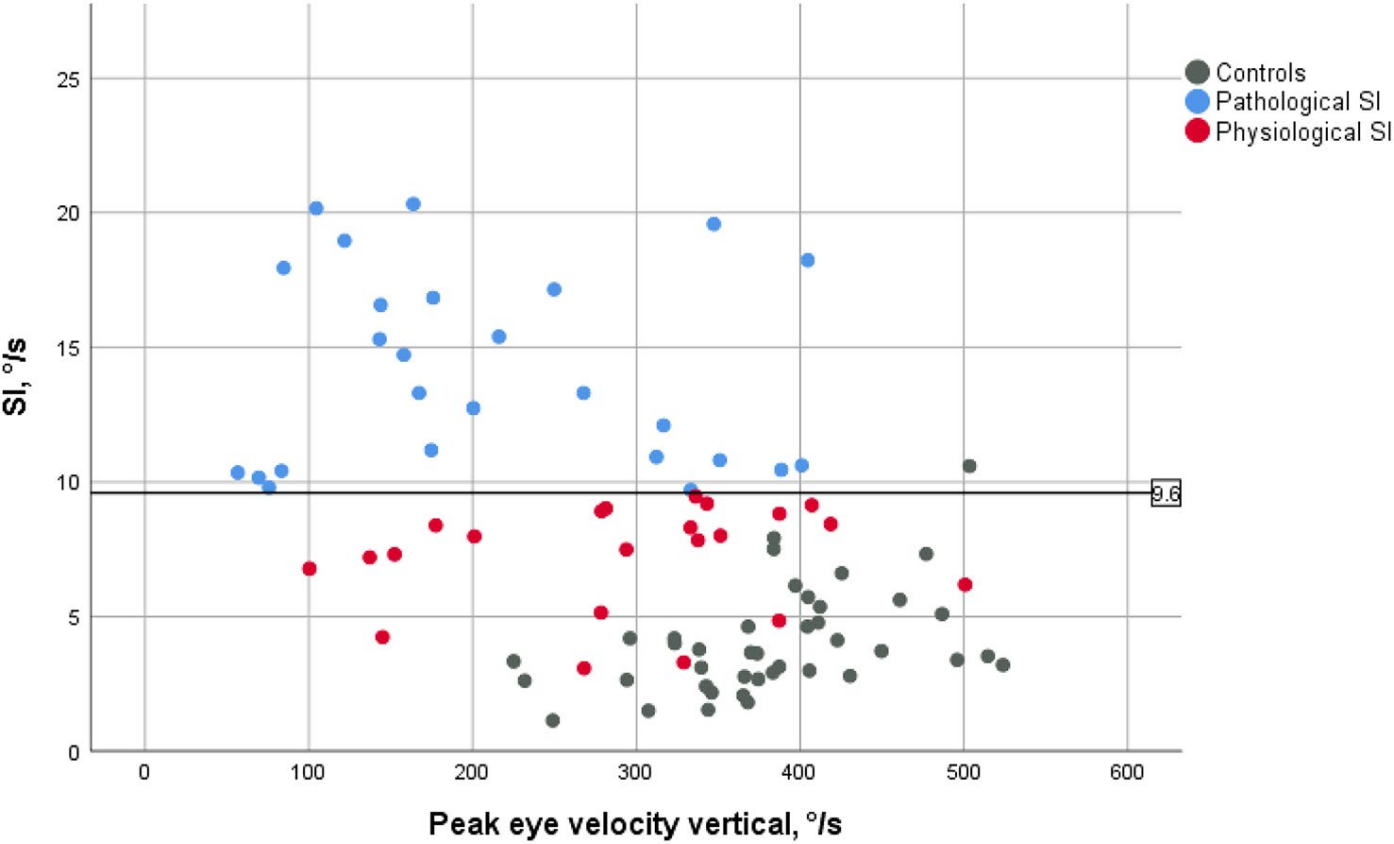
Rate of saccadic intrusions (SI) and the vertical peak eye velocity. The defined limit value for SI rate was 9.6°/s (black solid line). The rate of SI in patients defined as pathological (blue dots) was significantly higher than the rate reached by the control group (grey dots). Only few patients showed as few SI as the control group, and most of the patients defined as physiological with regard to SI (red dots) were in the upper range of the control group

For the SI rate, a value of 9.60°/s was determined as the limit for the classification into pathological and physiological values which corresponded to the 95th percentile of the age-corrected cohort of healthy controls. In 55 patients, measurements of the SI were available. 56.4% (*n* = 31) of the patients achieved higher, i.e., pathological, values, while 43.6% (*n* = 24) could be classified as physiological. As presented in Fig. 2, hardly any patients showed such a low SI rate as the control group. There was no correlation between SI rate and the value of vertical peak eye velocity.

### Vertical asymmetry

In order to objectively and reproducibly determine the absolute asymmetry *f* of the peak eye velocity in upward and downward direction, the following formula was used:

$$ f\left( {x,y} \right) = \left| {{\text{sgn}}\left( {x - y} \right)\left( {1 - \left( \frac{y}{x} \right)^{{{\text{sgn}}\left( {y - x} \right)}} } \right)} \right|, $$

with *x* is the peak eye velocity down in °/s and *y* is the peak eye velocity up in °/s.

**Fig. 3:**
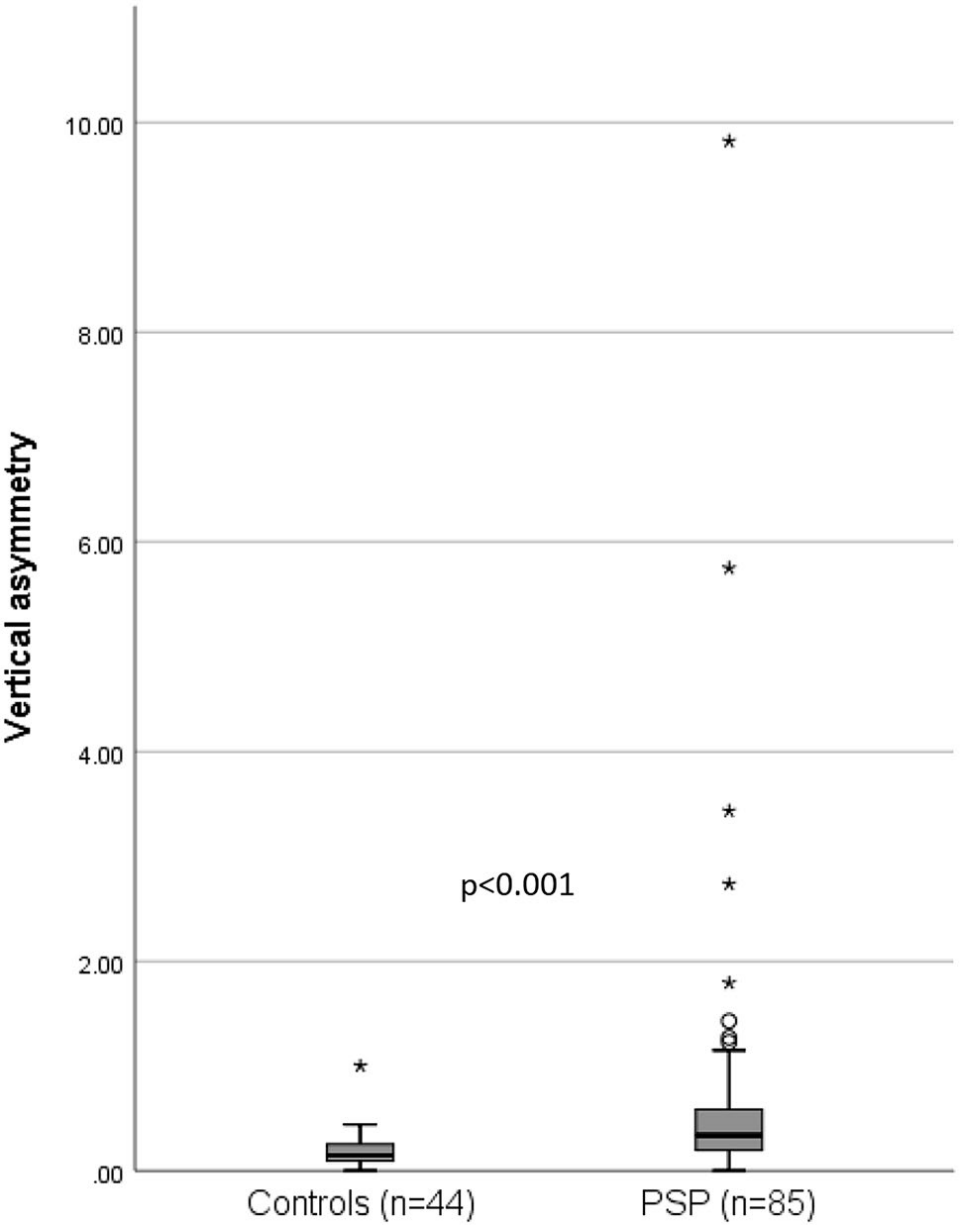
Measures of vertical asymmetry *f* of controls and PSP patients. The group difference with respect to vertical asymmetry was investigated by Mann–Whitney *U*-test for independent samples. *f* = 0 represents perfect symmetry and *f* = 1 implies one velocity is twice as much as the other

The whole patient cohort, as demonstrated in Fig. 3, as well as both subgroups PSP-RS and PSP-P separately, showed a significantly stronger vertical asymmetry than the control population (*p* < 0.001).

Among the controls, 56.8% (*n* = 25) showed an upward asymmetry, i.e., the velocity upwards was higher than the velocity downwards, while 63.5% (*n* = 54) of the patients showed a downward asymmetry, i.e., a higher velocity downwards. There was a significant difference between the distribution of asymmetry directions between patients and controls (*p* = 0.027). The asymmetry of the patients was more pronounced than that of the controls upwards and downwards, while a significant difference was found only downwards (*p* = 0.010). The variability of vertical asymmetry was higher among the patients.

The value of the 95th percentile of the vertical asymmetry of the control group represented an asymmetry of 0.421 (i.e., the velocity in one direction is about 1.4 times faster than in the other one), which was accordingly chosen as the cut-off value for the classification of symmetrical versus asymmetrical. Accordingly, 34.1% (*n* = 29) of the patients were considered asymmetrical; however, only two patients among those presented with physiological vertical peak eye velocities.

### Sensitivity and specificity of the oculomotor parameters

In the patient cohort, none of the oculomotor parameters showed a statistically significant correlation with the duration of the disease or the PSPRS score. The SI rate showed the best value (area under the ROC curve 0.938), a high specificity of 97.6%, and a moderate sensitivity of 54.2% by application of the defined limit value. The value of the vertical peak eye velocity was good (area under the ROC curve 0.858), the sensitivity based on the cut-off value was 56.5%, and the specificity was 95.5%. A limited value was achieved for the vertical asymmetry (area under the ROC curve 0.694) with a specificity of 95.1% determined by the cut-off value and a low sensitivity of 27.1%. Table 3 summarizes these results. In the separate analysis of the PSP-RS and PSP-P subgroups, there were slightly higher sensitivity values in the PSP-RS subgroup for most criteria, with a marked difference only for SI rate (63.0% vs. 46.2%); the only exception with a lower sensitivity was vertical asymmetry (23.9 vs. 44.4%). In the comparison of both subtypes, specificity was nearly identical. Table 4 summarizes the results separated for PSP-RS and PSP-P.

**Table 3:** Sensitivity and specificity of oculomotor parameters in the PSP group

	Area under the ROC-curve	Sensitivity (%)	Specificity (%)
VGRS (vertical) peak eye velocity, °/s	0.858	56.5	95.5
VGRS (up) peak eye velocity, °/s	0.880	62.4	95.5
VGRS (down) peak eye velocity, °/s	0.799	60.0	95.5
VGRS intrusion rate, °/s	0.938	54.2	97.6
VGRS vertical asymmetry	0.694	27.1	95.1

The calculation was performed by ROC analysis. The values given for sensitivity and specificity refer to the defined limit values

*VGRS *visually guided reactive saccades

**Table 4:** Sensitivity and specificity of oculomotor parameters separated for PSP-RS and PSP-P

	Area under ROC-curve (PSP-RS/PSP-P)	Sensitivity (PSP-RS/PSP-P)	Specificity (PSP-RS/PSP-P)
VGRS (vertical) peak eye velocity, °/s	0.895/0.825	58.7%/50.0%	95.5%/97.7%
VGRS (up) peak eye velocity, °/s	0.906/0.865	66.0%/55.3%	97.7%/95.5%
VGRS (down) peak eye velocity, °/s	0.842/0.717	60.4%/51.3%	95.6%/97.8%
VGRS intrusion rate, °/s	0.946/0.895	63.0%/46.2%	95.6%/95.6%
VGRS vertical asymmetry	0.695/0.771	23.9%/44.4%	95.5%/95.5%

The calculation was performed by ROC analysis. The values given for sensitivity and specificity refer to the defined limit values

*VGRS *visually guided reactive saccades

## Discussion

### Oculomotor deficits of PSP patients

To evaluate the value of specific oculomotor parameters, we analyzed video-oculographic recordings of 100 PSP patients and 49 age-matched healthy control subjects in this monocentric study. The correlates of oculomotor dysfunction parameters used for diagnosis of PSP according to the MDS proved to be abnormal in the patient cohort. More specifically, PSP patients had difficulty in performing smooth pursuit to track the target point at the required velocity so that the SPEM gain was reduced significantly. In a previous study on vertical SPEM, the gain of the PSP group deteriorated significantly with increasing frequency [19]. In another study, only the upward movements differed significantly in the vertical direction, whereas the horizontal SPEM differed in both directions, but still, the pronounced heterogeneity in the patients' values did not allow for a clear differentiation between individual patients and healthy controls [20]. In our study, the PSP patients performed inaccurate, hypometric reactive saccades whose amplitudes could not reach the size of the target amplitude. The vertical, especially the downward saccades, showed more pronounced deficits than the horizontal saccades. This hypometry of horizontal and downward directed saccades in PSP proved to be a suitable measurement value to differentiate between patients and controls at an individual level [20].

With respect to reduced peak saccade velocities, the differences between the patients and controls were highly significant in all directions, in line with previous studies [21, 22]. The quantitative difference was higher in the vertical than in the horizontal plane. The horizontal saccades have been shown to be affectated, but later in the disease course and to a lesser extent [11, 23, 24]. A detailed analysis of the vertical saccade velocities revealed larger reductions upwards than downwards as well as a significantly stronger asymmetry. It can be concluded that the upward saccades might be affected more frequently, but there are also studies in which no asymmetry of the vertical peak eye velocities was detected and reports of a primary impairment of the downward gaze [11, 12, 20, 25]. Thus, the findings are still heterogeneous in that respect.

The patient cohort showed a nearly threefold rate of SI compared to the control group, possibly because the total fixation movements increase which trigger reset movements [26]. Furthermore, PSP patients needed more time to initiate the execution of vertical and horizontal reactive saccades. These findings of a prolonged reaction time are in accordance with previous studies [27–29].

There are also similarities in oculomotor dysfunctions to other neurodegenerative parkinsonian syndromes, but the pronounced vertical saccade deceleration in PSP contrasts with the unchanged velocity values of Parkinson’s disease and multiple system atrophy [15, 16, 21]. This phenomenon allows for using the maximum vertical peak eye velocity for primary and differential diagnostics.

### VOG findings in the light of the oculomotor parameters of the MDS diagnostic criteria

When classified by the cut-off value of 240°/s for upward and downward primary saccade velocity, most patients showed pathological values. The fact that so many patients fulfilled the O2 criterion supports its use as a diagnostic criterion. However, there were also some patients with preserved saccade velocity. The determination of this VOG-based threshold allowed for the calculation of specificity and sensitivity. A high specificity of 95.5% was observed, in line with previous studies which however did not use an objective VOG approach [5], while the sensitivity was much lower at about 60%.

With a threshold of 9.60°/s, the SI rate of only about half of the patients was classified as pathological. However, given that about 20% of the patients with pathological SI rate had non-pathological velocities, the combination of vertical saccade velocities and the SI rate criterion increased the identification of PSP patients to almost 80%. The SI rate alone reached a sensitivity of 54.2% which is just below that of the vertical peak eye velocity, thus supporting the complementary function of this feature for diagnostic purposes. No correlation with disease duration or physical impairment could be found, similar to a previous study [20]. One reason for the somewhat unexpected lack of correlation with disease duration might be the rather homogeneous distribution of its values across the patient group. In the absence of a relation to disease progression, the suitability as a progression marker must be considered to be limited. However, with respect to the association with pathological changes in the brain, correlations between the reduction of the maximum vertical saccade velocity and macro- and microstructural as well as functional damage to the midbrain has been shown by advanced neuroimaging approaches [15, 16, 30, 31].

With a percentage of 55%, the classical entity PSP-RS was the most frequent subtype in the patient cohort, together with a relatively large number of patients with PSP-P (42%), along with only 3% of other clinical presentations. No oculomotor parameter could differentiate between PSP-RS and PSP-P. This finding is in line with a previous study which also did not find any differences in eye movements between both entities [20], confirming that the vertical peak eye velocities may also be affected in the early course of PSP-P. In the separate analysis of sensitivity and specificity in both subgroups, there were higher sensitivity values achieved for PSP-RS, while specificity was nearly identical. These findings are in a line of agreement with a recent VOG study in which PSP-RS patients only showed a significantly impaired downward smooth pursuit gain compared to PSP-P but otherwise no significant differences [32].

### Value of the asymmetry of vertical peak eye velocity

With a threshold of 0.421, one-third of the patients were classified to have asymmetric vertical peak eye velocities. The combination of this feature and maximum vertical peak eye velocity or SI did not increase the sensitivity. The specificity of the asymmetry alone of 95.1% was similar to the other parameters investigated, but it provided by far the lowest sensitivity of 27.1%, in the separate analysis of PSP-RS even only 23.9%. In summary, the asymmetry cannot be considered to be a useful addition to the current O-criteria.

### Limitations

The study was not without limitations. Since no post mortem neuropathological examinations of the brain are available for the patients in this study, no definitive diagnosis was possible. Furthermore, no follow-up examinations were included into the current analysis. Due to retrospective data acquisition, measurements of all parameters were not available for every patient, although there was a sizeable number of patients and control persons in each category. As a further limitation, the VOG data were acquired on two different commercial eye-tracking systems due to a change in the laboratory equipment. With the relatively high proportion of PSP-P compared to other cohorts, it must be taken into account that the distribution may not fully reflect clinical reality.

## Conclusion

In conclusion, the high value, especially the specificity, of the video-oculographically investigated maximum vertical peak eye velocities and SI rate based on data-driven thresholds resulted in support to the current form of use in the MDS-PSP diagnostic criteria as levels two and three in the oculomotor domain. Asymmetry of vertical velocities, on the other hand, could not be supported as a supplementary criterion. The fact that the oculomotor criteria alone—although they are the eponymous feature—were not sufficient to identify each and every patient is in line with the established procedure of the multifaceted syndromal diagnostic pathway in PSP, given that the diagnostic accuracy has gained further importance now that several therapeutic approaches, often directly targeting tau pathology, are being investigated in clinical trials [33].

## Supplementary Information

Below is the link to the electronic supplementary material.

Supplementary file1 (DOCX 15 KB)

## Data Availability

The data that support the findings of this study are available from the corresponding author upon reasonable request.
