# Supplementary material for: Diagnostic value of video-oculography in progressive supranuclear palsy: a controlled study in 100 patients
Source: J Neurol. 2021 Mar 21;268(9):3467–75. doi: 10.1007/s00415-021-10522-9 (PMC8357639; doi:10.1007/s00415-021-10522-9)
Supplement: Supplementary file 1 — Supplementary file1 (DOCX 15 KB) [file 415_2021_10522_MOESM1_ESM.docx]

**Supplemental Table 1 Oculomotor characteristics of PSP-RS and PSP-P** Data are presented as median, 95% confidence interval of the median (minimum - maximum value). ^a^Differences in the variables between groups were investigated by Mann-Whitney-U-test for independent samples. ^b^Presentation of the median of differences and the 95% confidence interval. VGRS = visually guided reactive saccades. SPEM = smooth pursuit eye movement.

|  | PSP-RS (*n*=55) | PSP-P (*n*=42) | *p* | Median of differences^b^ |
| --- | --- | --- | --- | --- |
| SPEM gain, % | 0.44; 0.33-0.51  (0.07-0.94) | 0.50; 0.42-0.61  (0.04-1.45) | 0.141^a^ | 0.06; -0.02/0.15 |
| VGRS latency, ms | 370.67;  325.60-395.79  (229.70-777.96) | 369.80;  310.27-419.79  (213.83-1020.70) | 0.593^a^ | -11.50;  -56.53/34.82 |
| VGRS (horizontal) gain, % | 0.72; 0.68-0.75  (0.18-1.12) | 0.73; 0.65-0.81  (0.20-1.05) | 0.406^a^ | 0.03; -0.04/0.1 |
| VGRS (up) gain, % | 0.50; 0.44-0.61  (0.03-1.07) | 0.54; 0.46-0.62  (0.03-0.96) | 0.942^a^ | 0.00; -0.10/0.11 |
| VGRS (down) gain, % | 0.55; 0.43-0.66  (0.05-1.05) | 0.65; 0.55-0.83  (0.12-0.98) | 0.118^a^ | 0.14; -0.01/0.25 |
| VGRS (horizontal) peak eye velocity, °/s | 291.29;  257.39-324.50  (130.63-747.31) | 321.26;  253.97-348.90  (162.22-596.66) | 0.347^a^ | 18.25;  -26.06/61.33 |
| VGRS (up) peak eye velocity, °/s | 173.31;  145.33-234.53  (58.91-477.78) | 211.80;  151.48-272.45  (54.07-450.53) | 0.572^a^ | 10.51;  -40.14/62.87 |
| VGRS (down) peak eye velocity, °/s | 206.01;  146.76-247.54  (13.15-573.29) | 232.11;  190.74-340.71  (49.83-571.99) | 0.157^a^ | 41.54;  -17.57/103.07 |
| VGRS intrusion rate, °/s | 11.19;  9.71-13.30  (3.29-20.16) | 9.19;  7.84-10.62  (3.08-23.20) | 0.109^a^ | -1.71; -4.93/1.14 |
